# Assessing the impact of extracellular matrix fiber orientation on breast cancer cellular metabolism

**DOI:** 10.1186/s12935-024-03385-3

**Published:** 2024-06-05

**Authors:** Madison R. Pickett, Yuan-I Chen, Mohini Kamra, Sachin Kumar, Nikhith Kalkunte, Gabriella P. Sugerman, Kelsey Varodom, Manuel K. Rausch, Janet Zoldan, Hsin-Chin Yeh, Sapun H. Parekh

**Affiliations:** 1 Department of Biomedical Engineering, The University of Texas at Austin, 107 W Dean Keeton Street Stop C0800, Austin, TX 78712 USA; 2 Centre for Biomedical Engineering, Indian Institute of Technology Delhi, New Delhi, 110016 India; 3 Department of Aerospace Engineering and Engineering Mechanics, The University of Texas at Austin, 78712 Austin, TX USA; 4 Department of Mechanical Engineering, The University of Texas at Austin, 78712 Austin, TX USA; 5 Oden Institute for Computational Engineering and Sciences, The University of Texas at Austin, 78712 Austin, TX USA; 6 Texas Materials Institute, The University of Texas at Austin, Austin, TX USA

**Keywords:** Extracellular matrix (ECM), Breast cancer microenvironment, Epithelial-to-mesenchymal transition (EMT), Cellular metabolism, Fluorescence lifetime imaging microscopy (FLIM), Optical redox ratio (ORR)

## Abstract

The extracellular matrix (ECM) is a dynamic and complex microenvironment that modulates cell behavior and cell fate. Changes in ECM composition and architecture have been correlated with development, differentiation, and disease progression in various pathologies, including breast cancer [1]. Studies have shown that aligned fibers drive a pro-metastatic microenvironment, promoting the transformation of mammary epithelial cells into invasive ductal carcinoma *via* the epithelial-to-mesenchymal transition (EMT) [2]. The impact of ECM orientation on breast cancer metabolism, however, is largely unknown. Here, we employ two non-invasive imaging techniques, fluorescence-lifetime imaging microscopy (FLIM) and intensity-based multiphoton microscopy, to assess the metabolic states of cancer cells cultured on ECM-mimicking nanofibers in a random and aligned orientation. By tracking the changes in the intrinsic fluorescence of nicotinamide adenine dinucleotide and flavin adenine dinucleotide, as well as expression levels of metastatic markers, we reveal how ECM fiber orientation alters cancer metabolism and EMT progression. Our study indicates that aligned cellular microenvironments play a key role in promoting metastatic phenotypes of breast cancer as evidenced by a more glycolytic metabolic signature on nanofiber scaffolds of aligned orientation compared to scaffolds of random orientation. This finding is particularly relevant for subsets of breast cancer marked by high levels of collagen remodeling (e.g. pregnancy associated breast cancer), and may serve as a platform for predicting clinical outcomes within these subsets [3–6].

**Supplementary Information:**

The online version contains supplementary material available at 10.1186/s12935-024-03385-3.

## Introduction

Breast cancer is the most common malignant tumor and the leading cause of cancer deaths among women globally [7]. It is known to exhibit strong metastasis, a marginally stable state wherein cells can leave primary tumors and initiate a new cellular mass (secondary tumor) in alternate locations in the body. During its metastasis, mammary epithelial cells (MECs) develop an invasive phenotype and depart from the primary tumor to spread into and colonize surrounding tissues and organs [8, 9]. Despite metastasis being the cause of 90% of cancer deaths, the precise cause for MEC transformation is yet known [10]; however, considerable research indicates that extracellular matrix (ECM) architecture plays a stimulatory role [11, 12].

The breast architecture is composed of MECs that make up acini connected to the ductal system [13]. Acini are surrounded by an ECM composed of a basement membrane composed of collagen IV and laminins immediately on their apical side followed by a fibrous stroma rich in fibrillar collagen I. These two systems work to biochemically and structurally support MEC function [1, 14–16]. Morphological and mechanical changes within the ECM microenvironment, specifically the increase in collagen fibril alignment and stiffness within the stroma have been linked to increased incidence of invasive breast cancer [2, 17]. For example, work by Chaudhuri et al. has shown that ECM mechanics and composition jointly impact MEC morphology and phenotype [16, 18]. Further studies have explored this observation through recapitulating breast ECM mechanics using hydrogel substrates [18–20]. However, these models do not contain the structural features found in the stroma of the ECM during invasion – namely, the dense fibrous architecture. In recent work, Riching et al., found that matrix topography rather than stiffness determined cellular invasion through 3D matrices, underscoring the importance of understanding the role of ECM architecture in cancer metastasis [21]. Synthetic electrospun nanofiber scaffolds have been used to better account for the structural properties of the ECM microenvironment in several cell motility studies [22, 23]. Specifically, polycaprolactone (PCL), a commonly used synthetic polymer, has been electrospun in several studies to mimic the ECM due to its benefits, which include biocompatibility, low cost, tunability, and ease of use [24–26]. These studies have demonstrated that an aligned architecture transforms MECs into invasive ductal carcinoma via the epithelial-to-mesenchymal transition (EMT), promoting motility and invasion. Changes in MEC invasiveness and phenotype have largely been quantified using cell area, motility, and expression of specific tumor markers and receptors such as overexpression of CD44, Snail and MMP2 [27–29]. However, studies focusing on the expression of individual tumorigenic markers and receptors fail to capture the upstream, broader cellular molecular signatures associated with changes in cell phenotype. Here, we focus on elucidating molecular signatures using single-cell imaging approaches to assess the effects of ECM orientation on breast cancer metabolism and outcomes.

It is well known that altered cellular metabolism is a hallmark of cancer progression and is characterized by enhanced glucose uptake and utilization to meet the energetic needs of metastasis [30]. Previous studies indicate that cancer cells have the ability to switch between different metabolic pathways in response to their microenvironments through a process known as “metabolic plasticity” [31]. Therefore, single-cell metabolic measurements are advantageous for understanding the energy-linked biological processes that drive phenotypic transformation and subsequent metastasis due to structural changes in the microenvironment. In this work, we use two label-free metabolic imaging methods of endogenous fluorophores: nicotinamide adenine dinucleotide (NAD(P)H) and flavin adenine dinucleotide (FAD^+^), for rapid and label-free characterization of cellular metabolic state [32, 33]. NAD(P)H and FAD^+^ are metabolic cofactors involved in generating ATP during oxidative phosphorylation, where NAD(P)H or NADH is oxidized to NAD^+^ and FAD^+^ is reduced to FADH_2_ [34]. Conveniently, NADH and FAD^+^ are endogenously fluorescent forms, allowing for visualization and quantification of the redox state of the cell in a label-free manner [35, 36].

Metabolic imaging of single cells is typically done with fluorescence lifetime imaging microscopy (FLIM) or two-photon excited fluorescence (TPEF). FLIM quantifies the fluorescent lifetimes of the (NADH and FAD^+^) endogenous fluorophores, where their lifetimes indicate the fractional component of the fluorophore in its protein-free and bound states. As a complementary technique, metabolic imaging can also be conducted using TPEF microscopy to measure intensities of cellular NADH and FAD^+^. Ratios of the intensities of these two endogenous fluorophores can be used to provide insight into the cellular redox state through a metric commonly known as the optical redox ratio, or ORR.

In this study, we aim to understand how changes in ECM orientation within a breast cancer microenvironment affect cellular metabolism. To accomplish this, we used biocompatible poly(caprolactone) (PCL) nanofiber scaffolds coated with a Matrigel layer to mimic the stromal tissue surround MECs in the breast. We used both “aligned” and “random” fiber orientations and evaluated single cell metabolic responses *via* both label-free FLIM and TPEF imaging of MECs two subtypes – a normal mammary epithelial cell line and a representative, highly invasive triple-negative cell line appropriate for in vitro studies [37]. For both MEC subtypes, we found that aligned fiber scaffolds caused cells to show a significant increase in a glycolytic metabolic phenotype compared scaffolds of random fiber orientation. Interestingly, cells also underwent a strong phenotypic transformation indicative of EMT progression on aligned scaffolds, possibly due to the known impact of the cellular microenvironment on cellular adhesive and migratory capacities [38].

## Methods

### Electrospinning and electrospun scaffold characterization

Nanofiber substrates of aligned and random orientation were fabricated by electrospinning of 10% w/v PCL (Sigma-Aldrich, 440744-250G) in Hexafluoro-2-propanol (Thermo Fisher Scientific, 147,541,000) onto a 7 cm rotating collector mandrel. Electrospinning parameters used for the fabrication of nanofibers were as follows: voltage 10 kV, solution flow rate 1.0 mL/h, needle gauge 24G, needle to collector distance of 11 cm, and collector drum rotating speed 500 rpm for random and 1000 rpm for aligned scaffolds. The morphology of electrospun fibers was evaluated using a scanning electron microscope (SEM). The orientation of the fibers was calculated using ImageJ/Fiji and MATLAB (Mathworks) to produce an orientation index based on Herman’s equations, where (S); S = 1 is fully aligned, whereas S = 0 is fully random [39]. Tensile testing of fiber mats was conducted on dogbone-shaped punchouts, where the neck of the sample was clamped, subjecting the gauge portion to mechanical testing (gauge width: 4.96 mm, gauge height: 5.00 mm, sample thickness: 0.06–0.16 mm), per ASTM standards [40]. Using an Instron device equipped with a 10 N load cell (Instron 5942, Norwood, MA), the sample was displaced at a rate of 0.2 mm/s and stretched up to 8 N. Displacement and normal force were recorded until sample breakage. Recorded stress, strain, and cross-sectional area of the sample were used to calculate Young’s modulus. Within the sample group, aligned or random, average sample thickness (ranging from 0.08 to 0.15 mm) was averaged for analysis. Aligned fibers were tested in both the parallel and perpendicular direction of fiber alignment, and a set of at least three scaffolds per condition was analyzed via mechanical testing.

### Cell culture

Cells (MCF-10 A and MDA-MB-231) were passaged at least two times after thawing before use. The cell lines selected were a highly invasive, metabolically adaptable, triple-negative (TNBC) cell line appropriate for in vitro studies, MDA-MB-231, and a normal mammary epithelial cell line, MCF-10 A. The MCF-10 A and MDA-MB-231 cell lines were selected to represent non-tumorigenic and metastatic states, respectively [41–44]. For all experiments, both cell lines were kept under passage number 30. MCF-10 A cells were cultured in Dulbecco’s Modified Eagle Medium (DMEM)/Ham’s F-12 1:1 with L-glutamine, HEPES (Cytiva Life Sciences, SH30023), supplemented with 5% heat-inactivated horse serum (ThermoFisher Scientific, 26,050,088), 1% Penicillin/Streptomycin Mixture (ThermoFisher Scientific BioReagents, BP295950), 10 µg/mL insulin (Sigma-Aldrich, I6634), 0.05% 1 mg/mL Hydrocortisone (Sigma-Aldrich, H0888), 0.02% 1 mg/mL epidermal growth factor (BioVision, 4022), and 0.01% 1 mg/mL cholera toxin Vibrio cholerae (VWR, 80,055). MDA-MB-231 cells were cultured in Dulbecco’s Modified Eagle Medium (DMEM)/ GlutaMax supplement with pyruvate, supplemented by 10% Fetal Bovine Serum (ThermoFisher Scientific, 26,140,079), 1% Penicillin/Streptomycin Mixture (ThermoFisher Scientific BioReagents, BP295950), and 10 µg/mL Insulin (Sigma-Aldrich, I6634). Cells were maintained in an incubator kept at 5% CO_2_ at 37^o^C.

Each scaffold was sterilized with 70% ethanol and UV light for one hour, and then washed with 1x sterile PBS twice. After sterilization, scaffolds were washed twice in DMEM and coated in Matrigel (Corning, CB-40,234) by incubating scaffolds in a 0.1 mg/mL Matrigel + DMEM only solution for 24 h before seeding. All scaffolds were coated in Matrigel due to their similarity to that of the MEC basement membrane and favorable properties for cell culture on biomaterial substrates [45]. Fourier transform infrared spectroscopy of pristine and Matrigel-coated PCL scaffolds confirmed successful coating (supplementary Fig. S1).

At 100% confluency, cells were lifted by washing with 1x sterile PBS and then incubating in Trypsin-EDTA Solution 0.25% (Caisson Labs, TRL01) and seeded on 1 cm^2^ square scaffolds at 50,000 cells per cm^2^. Each scaffold substrate was placed in an individual well within a 12 well plate, and one well within the plate was filled with 1x sterile PBS to maintain sample moisture. After cells were seeded on the scaffolds, they were allowed to adhere and grow for three days before imaging. During cell culture, each scaffold substrate was given 500 µL of media to cover the surface of the scaffold and prevent floating during culture. The cellular morphology of both cell lines on scaffolds was visualized using a Phalloidin and DAPI stain (supplementary Fig. S2).

### Immunofluorescence staining and quantification

Cells cultured on random and aligned scaffolds and were fixed with 4% paraformaldehyde after three days in culture. All samples were then washed twice with PBS and stored at 4 °C for no longer than 24 h before staining. Cells were then permeabilized with 0.5% Triton X-100 for 10 min and blocked with 3% bovine serum albumin for 30 min at room temperature. After blocking, cells were incubated overnight at 4 °C with the Alexa Fluor® 488 anti-Vimentin antibody (Biolegend, 699,305) at a concentration of a 1:300 dilution of the 0.5 mg/mL stock as detailed in the supplier manual. After vimentin labeling, the samples were counterstained for nuclei visualization using DRAQ5™ Fluorescent Probe Solution (ThermoFisher Scientific, 62,254) at a concentration of 10µM for two minutes before subsequent washing with PBS. After washing, scaffolds were mounted on No. 1 borosilicate 24 × 60 mm cover glasses and imaged (Vimentin ex/em: 499/520; DRAQ5™ex/em: 641/681) using an Olympus FV3000 confocal microscope and UPlanSApo 20x/0.75NA Olympus objective.

To quantify the immunofluorescence images, an automated pipeline was developed in CellProfiler to measure the integrated intensity of vimentin on a per-cell basis. Nuclei stained with DRAQ5 were identified as primary objects using global minimum cross-entropy thresholding. After identifying primary objects, propagation was used to locate the vimentin fluorescence as a secondary object and global minimum cross-entropy thresholding was used to determine the vimentin area. For each identified cell, the relative fold change of average vimentin intensity was calculated using the random condition as the baseline. Nuclear circularity was measured in ImageJ by manually identifying at least three regions of interest (ROI) per image using intensity thresholding of DRAQ5 fluorescence and the built-in circularity measurement.

### 2-NBD-Glucose uptake and quantification

2-NBD Glucose (2NBDG, ThermoFisher Scientific, N13195), a fluorescent glucose analog, was used to evaluate glucose uptake at the single cell level. Three biological and technical replicates of both MCF-10 A and MDA-MB-231 cells were seeded on scaffolds of aligned and random orientation at 50,000 cells/cm^2^ and allowed to adhere and proliferate for three days. After three days, the cells were washed with 1X PBS, and incubated 100 µM 2-NBDG in 1x PBS at 5% CO_2_ at 37^o^C. Cells on each scaffold condition were incubated for one hour before being washed twice with 1X PBS. After washing, all conditions were given phenol-free DMEM (ThermoFisher Scientific, 31,053,028) to maintain a favorable cellular environment prior to imaging. All conditions were imaged within at least an hour after being immersed in the phenol-free DMEM. For imaging, scaffolds were mounted on No. 1 borosilicate 24 × 60 mm cover glasses and imaged using an Olympus FV3000 confocal microscope and UPlanSApo 20x/0.75NA Olympus objective (2-NBDG ex/em: ~465/540 nm).

An automated pipeline was developed in CellProfiler to quantify the level of 2-NBDG fluorescence on a per-cell basis. Global thresholding was used to assist in segmenting cells within each image. From this, the pipeline evaluated integrated 2-NBDG intensity at the per cell level and these values were averaged per condition to achieve the results found in Fig. 2.

### **Real-time quantitative polymerase chain reaction (rt-*q*PCR)**

rt-*q*PCR was used to evaluate gene expression for vimentin, E-cadherin, Snail, CD44 and MMP2 on aligned and randomly oriented scaffolds. Three biological and technical replicates of both MCF-10 A and MDA-MB-231 cells were seeded on scaffolds of aligned and random alignment at 50,000 cells/cm^2^ and allowed to adhere and proliferate for three days. After three days, the scaffolds were immersed in lysis buffer and manually broken apart with tweezers in preparation for RNA extraction. RNA was extracted using the Quick-RNA Purification Kit, Miniprep, and Capped Columns (Zymo Research, R1054), according to the manufacturer’s protocol. Reverse transcription was completed using the High-Capacity cDNA Reverse Transcription Kit (ThermoFisher, 4,368,813), and the SYBR Green *q*PCR MasterMix (ThermoFisher Scientific, A46109) was prepared according to the manufacturer’s protocol. Quantitative PCR (*q*PCR) was performed using custom DNA Oligos (Millipore Sigma). *q*PCR was measured on a ViiA7 (Applied Biosystems), and relative expression levels were quantified using the 2^−ΔΔ*CT*^ method, using the Luna Universal qPCR Master Mix (New England Biolabs, cat no: M3003) manufacturers protocol. The full list of primers and primer sequences can be found in supplementary Table S1. For all conditions, ACTB (beta actin) served as the housekeeping gene [46].

### Fluorescence Lifetime Imaging Microscopy (FLIM) and data analysis

FLIM was used to quantify the NADH metabolic index. A FLIM system (Alba v5, ISS) was equipped with diode lasers providing the 405 nm lines, which passed through a multi-band dichroic mirror to excite samples. Fluorescence emission light traveled back along the same path to the multi-band dichroic mirror and went into avalanche photodiodes (APDs; SPCM-AQR-15, Perkin Elmer) with different bandpass filters (445/40nm, Semrock for NADH). A Nikon inverted microscope with 60x, NA 1.2 water objective (CFI60 Plan Apochromatic VC, Nikon) and an ASI XY automatic stage with motorized Z control were used in the current setup. The phase histogram was built up with the acquired photon counts using the FastFLIM unit [47]. The calibration in the FLIM system was done before imaging samples. A fluorescence lifetime standard, Atto 425, with a reference lifetime of 3.6 ns, was used as the calibration standard in this experiment. The laser repetition period used was 50 ns and was divided into 256 bins. FLIM images were scanned 3 times with a dwelling time of 0.1 ms/pixel. The data were collected by the confocal scanning system from a 256 × 256-pixel area (100 × 100 μm field of view) under different conditions.

The Fourier Transform was applied to the acquired fluorescence decay histogram to analyze the FLIM data in the digital frequency domain [48–50]. The fluorescence decay histogram of each pixel was converted into a specific phasor and then plotted as a single point in the phasor plot under the corresponding modulation frequency condition (*ω*_*k*_). The measured modulation ratios (*m*_*k*_) and phase shift (ϕ_*k*_) were then calculated from the phasor plots. The multifrequency modulation and phase data were analyzed using non-linear least squares in which the actual modulation ratio and phase were fitted to the bi-exponential decay model with two lifetimes for NADH. The two lifetimes had fractional contributions, *α*_*1*_ and *α*_*2*_ for the free and bound version of NADH, respectively. The NAD(P)H lifetime (Eq. 1) provides valuable insight into the redox state of the cell and can be quantified using the NADH metabolic index (Eq. 2) [51, 52].

1 $${\tau _{m,NADH}} = {\text{ }}{\alpha _1}{\tau _{1,}}_{NADH} + {\alpha _2}{\tau _{2,}}_{NADH}$$

2 $$NADH{\text{ }}Metabolic{\text{ }}Inde{x_ = }{\alpha _{1\left( {free} \right)/}}{\alpha _{2\left( {bound} \right)}}$$

Three biological and technical replicates of MCF-10 A and MDA-MB-231 cells were cultured at a density of 50,000 cells/cm^2^ on glass and on scaffolds of random and aligned orientation for three days. Cells cultured on the glass substrate were used as standards for processing subsequent FLIM measurements taken on the scaffolds.

### Two-photon imaging

Optical redox ratio (ORR) was used to evaluate the metabolism of both cell lines. An Olympus FV3000 confocal microscope was used to conduct two-photon measurements using an incorporated tunable femtosecond laser (Insight X3 Dual, Spectra-Physics). A UPlanSApo 20x/0.75NA Olympus objective was used to collect the autofluorescence signals of NADH and FAD^+^. NADH and FAD^+^ were measured at an excitation of 750 nm and 860 nm, respectively. Fluorescence emission was collected using a 495 nm dichroic to split and collect NADH using a 460±60 nm filter and FAD^+^ using a 530±43 nm filter (all from Thorlabs). The ORR value was calculated on a pixel-by-pixel basis per cell within each field of view as I_FAD+_/(I_FAD+_+I_NADH_). An ORR of 1 represents a shift towards a state of more oxidative phosphorylation and a value closer to 0 indicates a shift towards a more glycolytic state [53]. A 2.5 µM Rhodamine B solution in Ultrapure water was used to normalize images between settings. Live cells were imaged for less than 10 min after leaving the incubator to ensure cell health and viability during imaging. For each field of view, five cells were selected randomly and segmented to exclude the nucleus but included the entire cytoplasmic region using MATLAB.

### Statistical analysis

For all characterization experiments, at least 3 biological replicates (N), and two technical replicates (n) were analyzed per condition. Statistical differences were found using the statistical tests detailed in figure captions using GraphPad Prism software.

## Results

### Aligned and randomly oriented nanofiber scaffold characterization

**Fig. 1:**
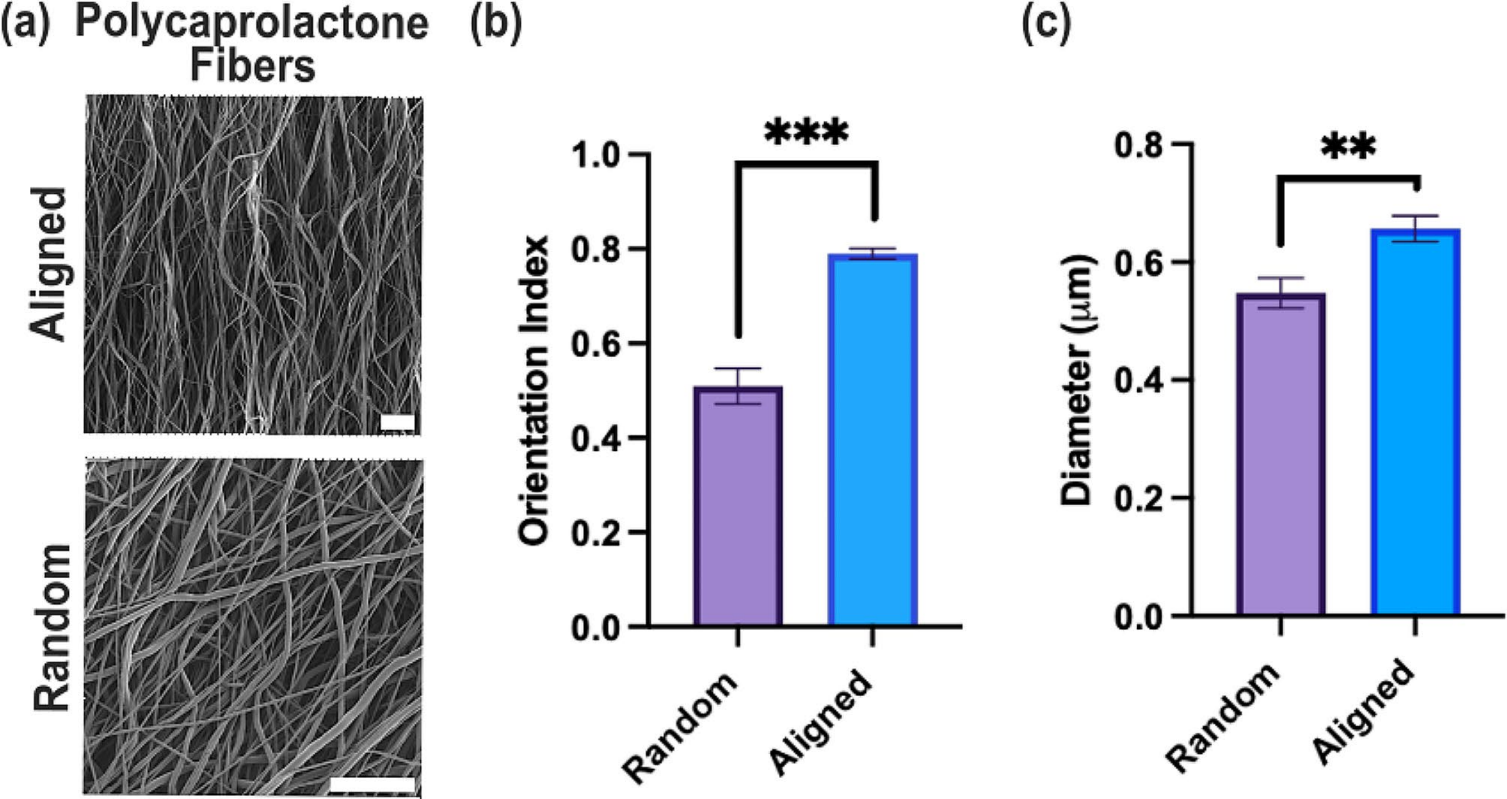
**(a)** Random and Aligned Polycaprolactone electrospun nanofibers were assessed for **(b)** orientation index and **(c)** fiber diameter. Scale bar: 15 μm

We evaluated the metabolic signatures of the MCF-10 A (normal MEC) and MDA-MB-231 (triple-negative, invasive MEC) cell lines on Matrigel-coated aligned or randomly oriented electrospun nanofiber substrates as ECM stroma mimics of the breast. Electrospun scaffolds of PCL (random and aligned) were characterized for nanofiber orientation, diameter, and stiffness. Orientation analysis resulted in significant differences between the orientation indices, represented by S, for aligned (S = 0.79±0.01) and random (S = 0.51±0.38) nanofibers. The orientation indices were selected to mimic the architecture of collagen fibers in the stroma before (random) and during (aligned) tumor metastasis [54–56]. Fiber diameter was also significantly different between the two scaffold orientations, with a mean aligned diameter of 0.46±0.02 μm and a diameter of 0.54±0.03 μm for the random condition (Fig. 1). Fiber diameter is known to decrease with increased rotational velocity of the rotating mandrel, accounting for the difference in fiber diameter between random and aligned conditions [57]. However, our difference in fiber diameter (0.08 μm) is less than the standard deviations often reported in literature [24, 58, 59]. Therefore, we believe the difference in nanofiber diameter has a negligible effect on cellular outcomes. The aligned and the random scaffold had Young’s moduli of 1.39±0.16 MPa and 0.88±0.11 MPa, respectively, indicating a higher substrate stiffness for the aligned condition.

The aligned condition tested perpendicular to alignment was the least stiff of all groups tested, with a stiffness of 0.75±0.06 MPa. All young’s moduli were consistent with reported values for PCL fiber mats [60, 61]. While the difference in stiffness between sample conditions is significant, all values remain well above a physiologically relevant Young’s modulus (~ 15.0 kPa, ex vivo invasive ductal carcinoma) and the upper sensing limit for breast cancer mechanotransduction pathways reported in literature (~ 10–100 kPa) [10, 62–64].

### Quantifying glucose uptake

**Fig. 2:**
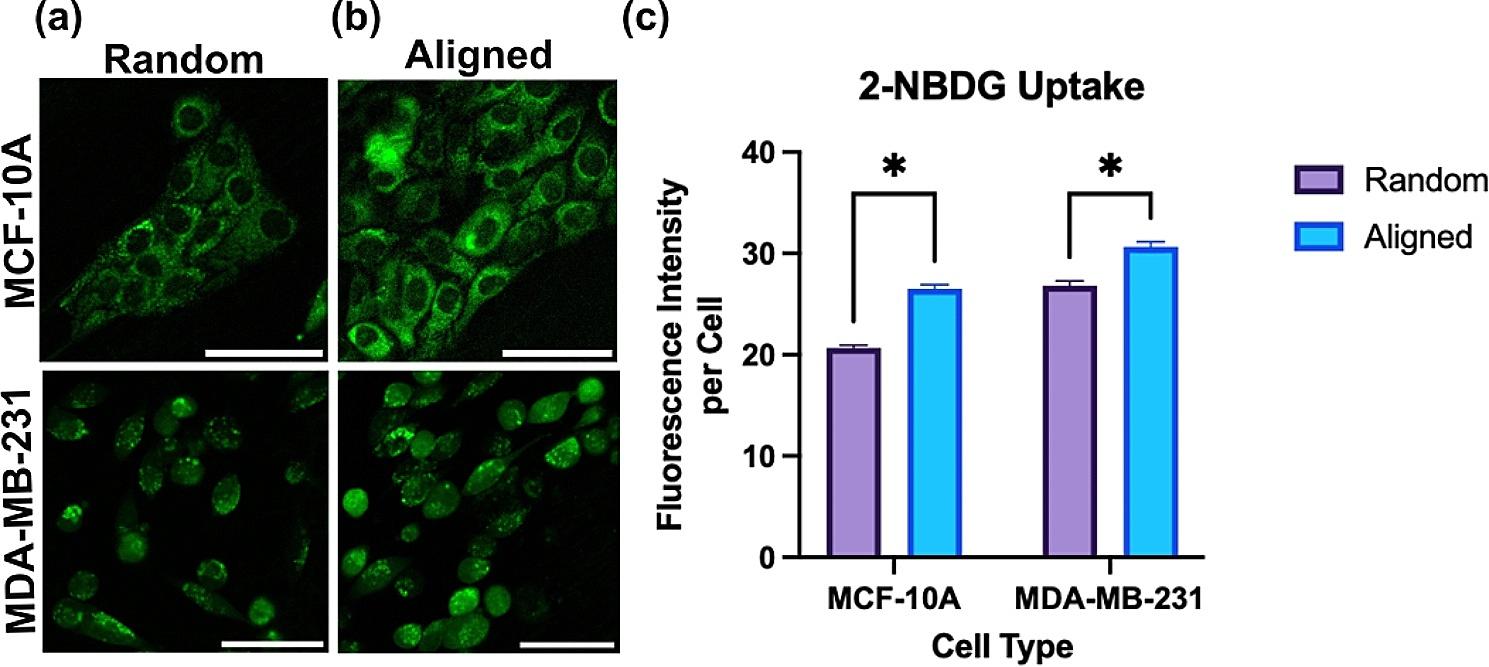
Fluorescence intensity images of MDA-MB-231 and MCF-10 A cells on **(a)** random and **(b)** aligned scaffolds. Green: 2-NBDG; scale bar: 50 μm. **(c)** Quantified 2-NBDG intensity per cell for MCF-10 A and MDA-MB-231 cells (*p < 0.05) on both scaffold conditions by Welch’s t-test. Three biological (*N* = 3) and technical replicates (*n* = 3) were measured for each condition. Error bars represent the standard error of the mean

Understanding glucose uptake is integral to understanding metabolism, as glucose is the precursor for several metabolic pathways, including glycolysis [65]. 2-NBDG, a fluorometric glucose analog, is a reliable way to assess glucose uptake at the single cell level [66]. After an hour incubation with 2-NBDG, live cell imaging was conducted for both cell lines on both scaffold conditions. After imaging, a pipeline in CellProfiler was used to determine integrated intensity at the single cell level. These values were averaged for each condition, as reported in Fig. 2. For both scaffold conditions, the MDA-MB-231 cell line demonstrated a greater fluorescence intensity than that of the MCF-10 A cell line. This is consistent with prior findings, as the triple-negative, highly invasive MDA-MB-231 cell line is inherently more glycolytic and therefore takes up more glucose [67]. Additionally, we observed increased 2-NBDG fluorescence intensity per cell on aligned scaffolds compared to random scaffolds for both MCF-10 A and MDA-MB-231 cell lines, indicating increased glucose uptake in the aligned microenvironment for both cell lines.

### Fluorescence lifetime metabolic imaging of NADH

With changes in 2-NBDG uptake suggestive of adapting metabolic demands for both normal and invasive MECs in response to nanofiber scaffold alignment, we sought an alternative method to elucidate shifts in cellular metabolism. To accomplish this, we imaged changes in the cellular metabolism of MDA-MB-231 and MCF-10 A cells in response to scaffold alignment through FLIM measurements of NADH. The fluorescence lifetime of NADH consists of two components: a shorter lifetime (~ 0.4 ns) when free in the cytosol and a longer lifetime (spanning from ~ 1.4 ns to 9 ns) when protein-bound [53, 68, 69]. An increase in the mean NADH lifetime – suggestive of a larger percentage of bound NADH – indicates a more oxidative metabolism while the inverse indicates a metabolism undergoing more glycolysis. The ratio of free to bound NADH can be characterized *via* the NADH metabolic index (Eq. 2 above), which reflects changes in metabolism on the single-cell level.

**Fig. 3:**
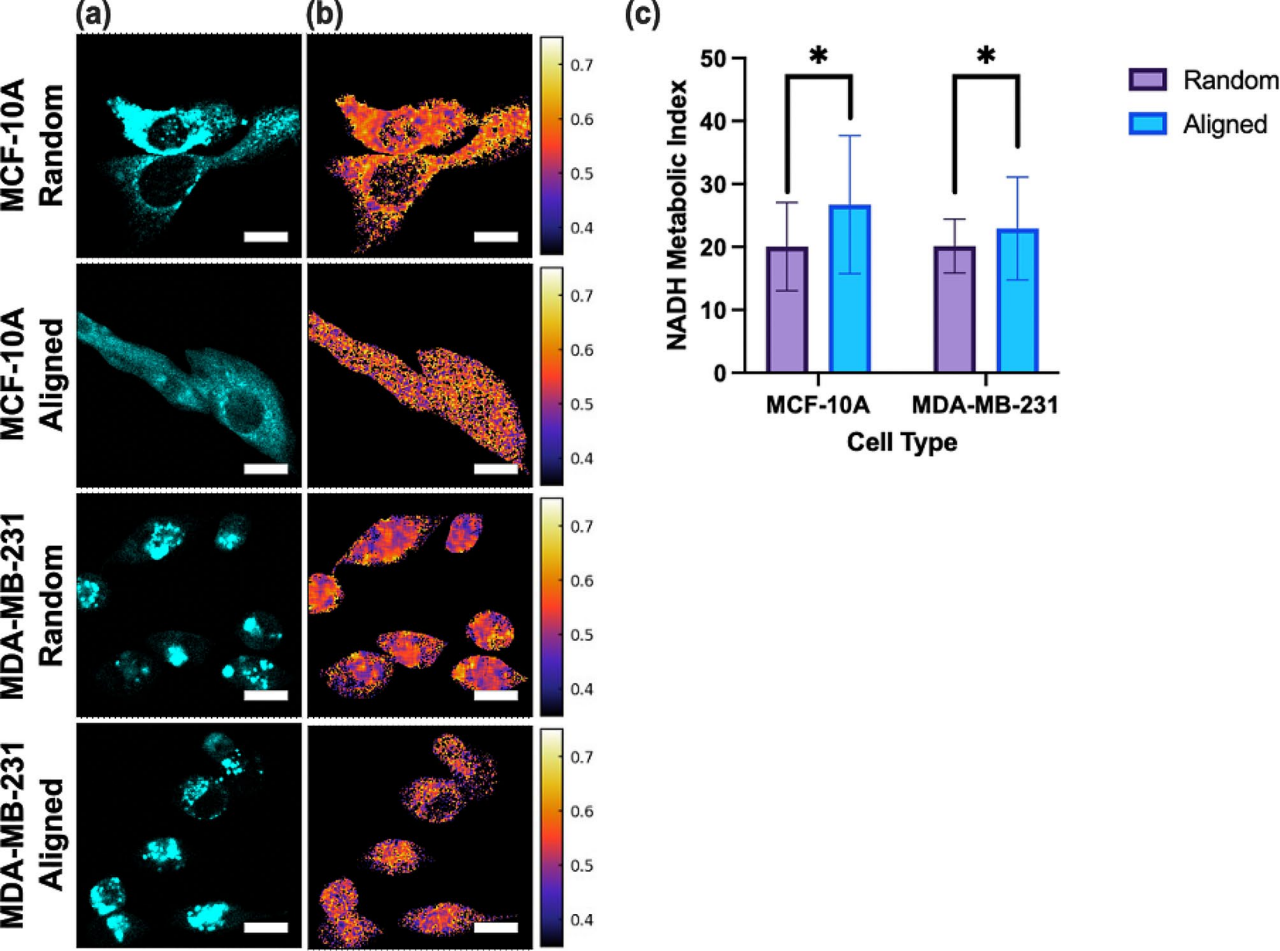
**(a)** Intensity contrast and **(b)** FLIM images of NADH for MDA-MB-231 and MCF-10 A cell lines on random and aligned Matrigel conditions, color bar representing average NADH lifetime value. Scale bar: 20 μm. **(c)** Bar graphs of NADH metabolic index for the MDA-MB-231 and MCF-10 A cell lines on random and aligned Matrigel conditions (error bars, standard deviation, three biological replicates (*N* = 3), ten cells per condition analyzed (*n* = 10)). *p < 0.05 using the Kolmogorov-Smirnov test

Both highly invasive MDA-MB-231 cell line and MCF-10 A non-tumorigenic epithelial cells were seeded on random and aligned nanofiber scaffolds. For both the MDA-MB-231 and MCF-10 A cell lines, a significant difference in NADH metabolic index was observed for cells on the aligned versus random condition (Fig. 3) [70, 71]. On their respective aligned scaffolds, MDA-MB-231 demonstrated a 13.89% increase and MCF-10 A a 33.18% increase in NADH metabolic index (Fig. 3c). Both changes indicate that alignment drives a more glycolytic phenotype.

### Two-photon intensity metabolic imaging

Two-photon, intensity-based imaging was used to confirm the metabolic signature trends observed in the FLIM results. Previous reports have shown that it is possible to differentiate and quantify metabolic trends at the single-cell level using a ratio of the endogenous fluorescence intensities of NADH and FAD^+^, known as the optical redox ratio (ORR) [72, 73]. We therefore used the ORR as an additional measure of the metabolic activity of the cells on both scaffolds. The ORR was calculated as I_FAD+_/(I_FAD+_+I_NADH_), with a decrease in ORR indicating an increase in glycolytic activity [74, 75].

**Fig. 4:**
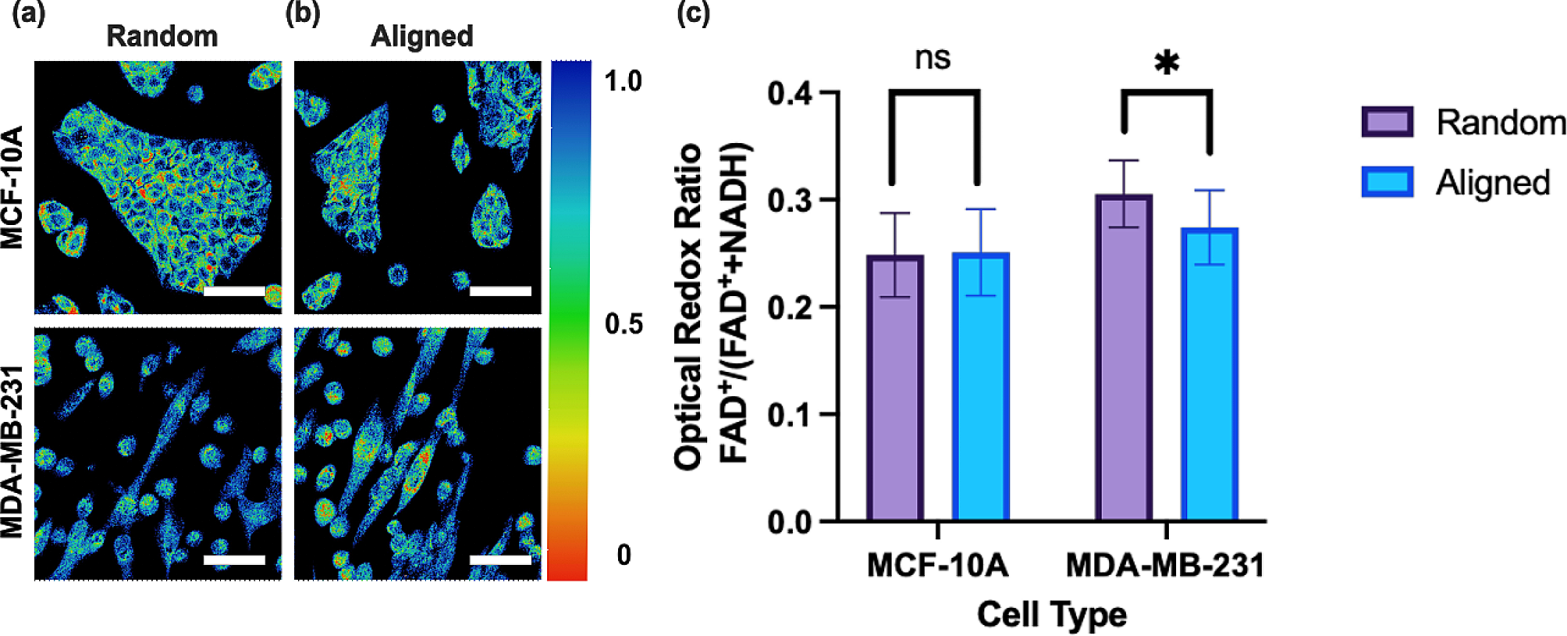
Two-photon intensity images of MDA-MB-231 and MCF-10 A cells on **(a)** random and **(b)** aligned Matrigel-coated scaffolds. Images processed to represent ORR of FAD^+^/(FAD^+^+NADH). Scale bar: 50 μm **(c)** Normalized ORR values for MCF-10 A (*p* = 0.438) and MDA-MB-231 cells (***p < 0.001) on scaffold conditions using the Kolmogorov-Smirnov test. Error bars represent the standard error of the mean. All ORR data were quantified at the whole-cell level, followed by segmentation to exclude the nucleus, as well as the background for each condition during processing [45]. For visualization purposes, the background in the ORR images in Fig. 5 was subtracted. All conditions had a sample size of (*N* = 4, *n* = 3, 5 cells per field of view analyzed)

Quantitatively, the mean ORR of MDA-MB-231 cells was 13.79% lower on aligned scaffolds (0.27±0.03) than on the randomly oriented scaffold (0.31±0.03), indicative of a shift toward a glycolytic metabolism for the MDA-MB-231 cells on the aligned condition (Fig. 4c). However, for the MCF-10 A cell line, no significant change in mean ORR was observed between the aligned (0.25±0.04) and random (0.25±0.04) scaffold condition (Fig. 4c). Findings from the FLIM studies suggest that an aligned scaffold orientation drives a more glycolytic phenotype for both cell lines, and TPEF results support this finding for the MDA-MB-231 cell line.

### Investigating the epithelial-to-mesenchymal transition and cellular alignment

**Fig. 5:**
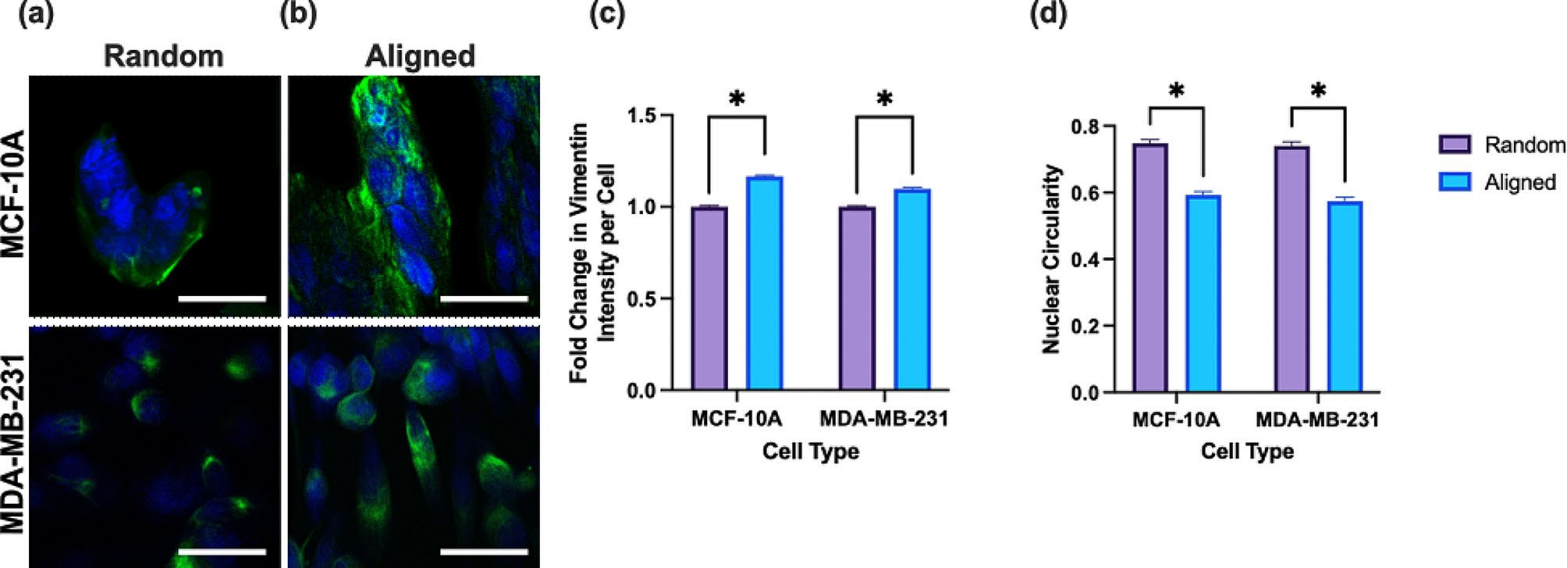
Fluorescence intensity images of MDA-MB-231 and MCF-10 A cells on **(a)** random and **(b)** aligned scaffolds. Blue: Draq5 nuclei stain, Green: Vimentin Stain. **(c)** Normalized Vimentin Intensity **(d)** Nuclear circularity per cell for MCF-10 A and MDA-MB-231 cells (*p < 0.05, ***p < 0.001) on both scaffold conditions by student’s t-test. Error bars represent the standard error of the mean. Scale bar: 100 μm. Sample sized analyzed was as follows; aligned MDA-MB-231 (three biological *N* = 3, two technical *n* = 2, 370 cells analyzed), random MDA-MB-231 ( three biological *N* = 3, three technical *n* = 3, 514 cells analyzed), aligned MCF-10 A (three biological *N* = 3,three technical *n* = 3, 180 cells analyzed), random MCF-10 A (three biological *N* = 3, three technical *n* = 3, 109 cells analyzed)

Metabolic reprogramming is associated with the epithelial to mesenchymal transition (EMT) during metastasis. The EMT is a process in which epithelial cells adopt more mesenchymal properties, advantageous for invasion and migration [76]. Therefore, to investigate the EMT, we looked to measure vimentin, a protein involved in cell adhesion and stability as well as EMT progression, using immunofluorescence staining and confocal imaging. MCF-10 A and MDA-MB-231 cells were seeded on aligned and random electrospun scaffolds, respectively, and allowed to grow for three days before fixation and staining. Both cell lines contain vimentin intermediate filaments and are therefore widely used to investigate changes in vimentin expression during EMT progression [77–79]. Vimentin expression was quantified at the single-cell level using a pipeline developed in CellProfiler [80]. Integrated intensity values of vimentin fluorescence per cell were used to calculate the relative fold change in vimentin intensity between aligned and randomly oriented scaffolds. For both the MDA-MB-231 and MCF-10 A cell lines, the aligned scaffold condition exhibited significantly higher vimentin expression than the random condition. The average vimentin expression per cell for MDA-MB-231 cells was 13% higher for the aligned condition than for the random condition with *p* < 0.001 MCF-10 A cells showed an 11% increase in vimentin expression on the aligned scaffold material compared to the random scaffold with a *p* = 0.025 (Fig. 5c).

To further investigate cellular response, we analyzed nuclear shape as another indicator of cell response to fiber alignment by measuring the nuclear circularity. Nuclear circularity has been seen to significantly impact cell proliferation, migration, and metastasis [81, 82]. DRAQ5 fluorescence was used to identify the region of interest and was used to determine nuclear circularity as $Circularity=4\pi (area/{perimeter}^{2})$. A value of 1 indicates a shape that is a perfect circle. Both cell lines were found to have significantly different nuclear circularity between scaffold conditions, with values closer to 1 on the random scaffolds. MCF-10 A average nuclear circularity was 0.59±0.01 for the aligned scaffolds and 0.75±0.01 for the random scaffolds (Fig. 5d). MDA-MB-231 cells showed similar trends with an average nuclear circularity of 0.58±0.01 for the aligned condition and 0.75±0.01 for the random condition (Fig. 5d). The decreased nuclear circularity for the aligned scaffolds suggests changes in cellular alignment are due to the orientation, and possibly mechanical changes, of the aligned scaffolds. Aligned fibers have been reported to trigger intracellular signaling to increase the vimentin expression, or more mesenchymal phenotype on aligned substrates [83]. In agreement with the reported literature, our results support that aligned scaffolds promote both an increase in the vimentin expression and elongated nuclear morphology, characteristic of EMT progression [83–85]. Additionally, we used 488 Phalloidin to visualize actin filament organization on scaffolds of both random and aligned orientation for both cell lines, as seen in Figure S2. Qualitatively, cells appeared to be more clustered on PCL scaffolds when compared to glass substrates. Additionally, the MDA-MB-231 cells appeared to take on a more elongated morphology on the aligned PCL substrate when compared to the glass and random PCL substrate.

**Fig. 6:**
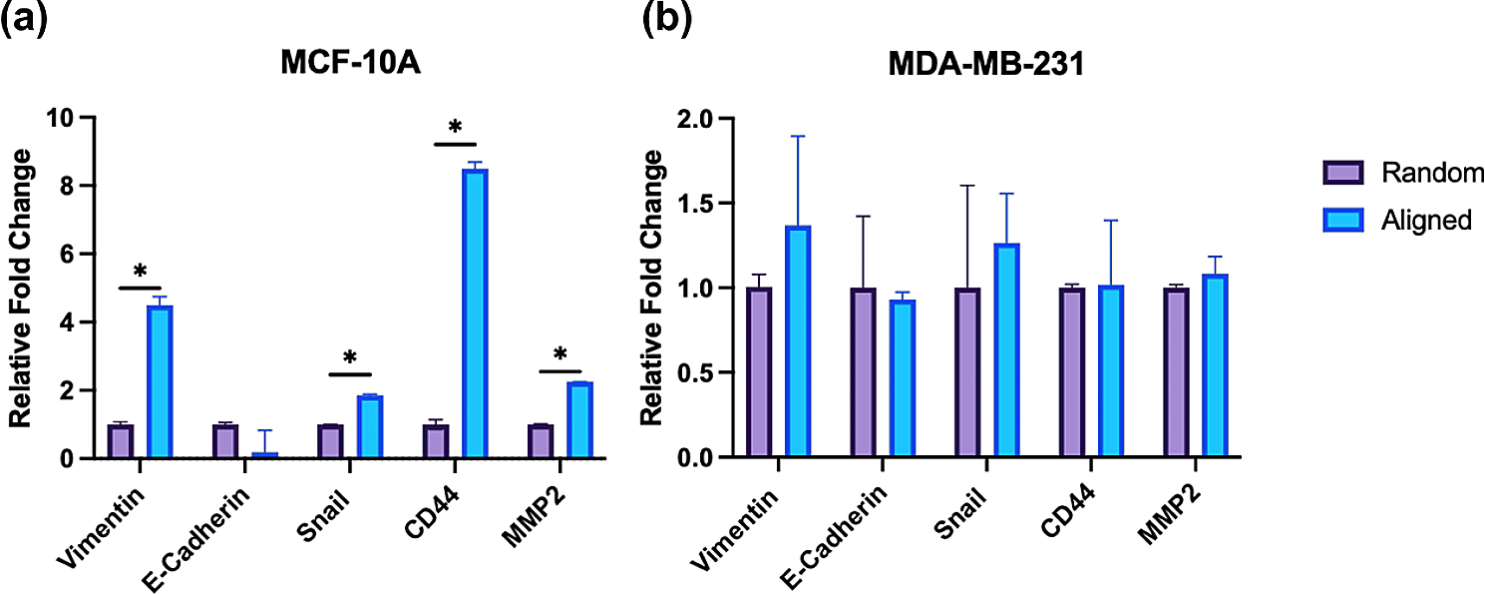
rt-*q*PCR bar graphs of relative normalized RNA expression of vimentin, E-cadherin, Snail, CD44 and MMP2 for **(a)** MCF-10 A and **(b)** MDA-MB-231 cells on random and aligned polycaprolactone electrospun nanofibers. (*p < 0.05) by t-test. Error bars represent the standard error of the mean. For all scaffolds conditions three biological *N* = 3 and two technical *n* = 2 repeats

To further examine EMT progression, we turned to rt-*q*PCR to assess EMT-associated gene expression. Vimentin and E-cadherin are known markers of the EMT, which is marked by the loss of intracellular junctions (E-cadherin) and increased expression of vimentin. Increased vimentin expression was observed on aligned scaffolds for both cells lines, albeit only significant for the MCF-10 A cell line (Fig. 6a). For both cell lines there was no significant difference in E-cadherin expression between scaffolds of random and aligned orientation. However, there was a reported decrease in E-cadherin expression for both cell lines on the aligned scaffold condition. To further investigate the impact of scaffold orientation on tumor progression, we also measured the expression levels of Snail, CD44 and MMP2 (Matrix Metalloproteinase-2). All three of these markers are known promoters of the EMT, suggesting that increased expression indicates a more mesenchymal or invasive cellular phenotype [27, 28, 86, 87]. We found that expression for all three markers was significantly increased for the MCF-10 A cell line on aligned scaffolds: Snail (1.86-fold), CD44 (8.48-fold) and MMP2 (2.25-fold) (Fig. 6a). However, for the MDA-MB-231 cell line no markers were significant between random and aligned conditions (Fig. 6b). Nevertheless, our findings suggest that changes in extracellular microenvironment alignment, from S = 0.58 to S = 0.75, can induce EMT progression in the normal mammary epithelial MCF-10 A cell line.

## Discussion

This study investigated the effect of stromal alignment on cellular metabolic signatures and epithelial-to-mesenchymal transition (EMT) phenotypic transformation using an engineered stromal environment of Matrigel-coated nanofibrous scaffolds. Previous works have linked changes in collagen density and stiffness to changes in cellular metabolism and EMT progression, yet no studies have examined the impacts of ECM orientation on single-cell metabolic outcomes [62, 88]. Both stiffer and aligned ECM microenvironments have been reported to influence cell motility and phenotype to aid cancer cells in invasion and metastasis [18, 22, 89]. Therefore, we hypothesized that cells grown in a more aligned microenvironment would show a metabolic signature and phenotype matching well with that of invasive cancer. To investigate our hypothesis, nanofiber scaffolds were electrospun in random and aligned orientations and coated with Matrigel to mimic the protein composition and structure of the breast ECM seen in normal and invasive BC. Two metabolically distinct breast cell lines were seeded on the scaffold conditions: a normal mammary epithelial cell line, MCF-10 A, and a highly invasive, metabolically adaptable, triple-negative (TNBC) cell line appropriate for in vitro studies, MDA-MB-231. Changes in specific metabolic and EMT markers were quantified using immunofluorescence and rt-*q*PCR between scaffold conditions, whereas direct single-cell metabolic imaging was used to provide a more robust understanding of cellular metabolic outcomes caused by ECM fiber orientation in stromal tissue mimics.

To begin investigating alignment effects, we looked to 2-NBDG uptake to provide insight on glucose uptake and larger metabolic trends. For both cell lines, 2-NBDG uptake was significantly higher on the aligned scaffold condition compared to the random scaffold condition, suggesting increased glucose consumption for cells within the more aligned microenvironment. Since glucose utilization is indicative of glycolysis, a metabolic pathway linked to metastasis, we looked to alternative methods to quantify shifts in cellular metabolism due to alignment effects on a broader scale. This was accomplished using lifetime and intensity approaches to quantify single-cell metabolic states. FLIM measurements of NADH suggested that variations in alignment of the cellular microenvironment resulted in significant differences in cellular metabolic signatures. We found that the average fluorescence lifetime values of NADH in both cell lines were consistent with those in the literature [90, 91, 92]. For both cell lines, we observed a decrease in average NADH lifetime for cells on the aligned condition as quantified by the NADH metabolic index. This result indicates an increase in free cellular NADH and is associated with increased glycolysis compared to oxidative phosphorylation, supporting our hypothesis that cells within a more aligned microenvironment adopt a more glycolytic metabolic signature [23, 27, 41–43].

We also used TPEF to image NADH and FAD^+^ endogenous fluorescence intensity from cells within the scaffold material as a complementary method to FLIM due to the increased light penetration depth capabilities [93]. Two-photon intensity images were collected for each respective coenzyme, and the ORR, defined as I_FAD+_/(I_FAD+_+I_NADH_), was reported per cell. The ORR results for MDA-MB-231 cells were consistent with the metabolic conclusions derived from FLIM, indicating a significant shift towards glycolysis on the aligned scaffold condition. ORR for MCF-10 A cells was not statistically significant. This discrepancy could arise from interfering flavin mononucleotide (FMN) endogenous fluorescence, a cofactor associated with oxidative phosphorylation but is not involved in aerobic glycolysis [94]. FMN is spectrally similar (both in excitation and emission) to FAD^+^ and can thus interfere with the pure FAD^+^ signal [36, 95]. FLIM, which uses lifetime analysis of NADH autofluorescence to make conclusions about the metabolic state, does not require FAD^+^ measurements and, therefore, avoids potential FMN interference.

For a more functional interpretation of our metabolic findings, we looked at changes in EMT markers for each cell type as EMT is a process that plays an integral role in cancer invasion and metastasis [34, 96]. EMT progression was quantified using immunofluorescence to measure vimentin, a type III intermediate filament [97]. In invasive ductal carcinomas, vimentin is highly expressed and correlated with increases in metastatic potential, specifically increases in invasion, migration, and therapeutic resistance. Both cell lines demonstrated significantly higher vimentin protein expression on the aligned, Matrigel-coated nanofiber scaffolds than on the random, Matrigel-coated scaffolds. To further quantify phenotypic changes resulting from changes in alignment, we used immunofluorescence data to measure nuclear circularity for both conditions. We found that nuclei in both cell lines on the aligned condition adopted a more elongated phenotype, indicating a higher degree of progression through the EMT [98].

To confirm initial immunofluorescence findings, we used rt-*q*PCR to measure markers of EMT progression for both cell lines. Increased expression of vimentin, Snail, CD44 and MMP2 are correlated with increased invasiveness, whereas decreased levels of E-cadherin indicate EMT progression [27, 28, 87, 99, 100]. For the normal MCF-10 A cell line, vimentin, Snail, CD44 and MMP2 expression were all significantly higher on the aligned scaffold compared to the random scaffold. Changes in E-cadherin expression between scaffold conditions was not significant, however, average expression decreased (0.19-fold), indicative of EMT progression. The highly invasive, triple negative MDA-MB-231 cell line demonstrated no significant differences in expression levels for any gene between scaffold conditions as these cells are more mesenchymal by nature. While several markers reported changes consistent with expected observations during EMT progression, none were large enough to substantiate a statistically significant invasive transformation of the MDA-MB-231 cell line. We believe this is due to the inherent nature of the subtype. MDA-MB-231 is often used as a representative mesenchymal subtype when investigating the inverse of the EMT, the mesenchymal-to-epithelial transition (MET) [101]. Therefore, at baseline, EMT markers are highly upregulated for the MDA-MB-231 cell line, making it difficult for changes in expression levels to be detected by bulk measurements like rt-*q*PCR [102].

## Conclusion

In conclusion, our study reveals some underlying mechanisms that the ECM triggers for both normal and invasive breast epithelial cells. While prior art has investigated the effects of stromal orientation on breast cancer metastasis in tissue samples, no studies have employed a biomaterial substrate to study how ECM fiber orientation effects on single-cell metabolic outcomes [21, 103–105]. Our system allowed us to isolate biomechanical changes of the ECM during metastasis, and study cellular outcomes without any of the confounding variables inherent to tissue/animal derived samples. Both normal and TNBC cells appear to show metabolic plasticity – that is, the ability for their metabolism to be influenced – solely by the stromal architecture in the ECM. The aligned stromal layer induces not only phenotypic changes in cells toward increased invasion, as shown previously, but also metabolic reprogramming to a more glycolytic phenotype consistent with invasive disposition. We believe label-free, single-cell metabolic imaging methods combined with biomimetic substrates are an excellent platform to further investigate the impact of tumor microenvironment on cell phenotypic outcomes.

### Electronic supplementary material

Below is the link to the electronic supplementary material.

Supplementary Material 1

## Data Availability

The materials and data that support the findings of this study are available from the authors on request.
