# Supplementary material for: Assessing the impact of extracellular matrix fiber orientation on breast cancer cellular metabolism
Source: Cancer Cell Int. 2024 Jun 5;24:199. doi: 10.1186/s12935-024-03385-3 (PMC11151503; doi:10.1186/s12935-024-03385-3)
Supplement: Supplementary file 1 — Supplementary Material 1 [file 12935_2024_3385_MOESM1_ESM.docx]

**Supplementary Information**

Assessing the impact of extracellular matrix fiber orientation on breast cancer cellular metabolism

*Madison R. Pickett^1^, Yuan-I Chen^1^, Mohini Kamra1, Sachin Kumar^1,2^, Nikhith Kulkunte^1^, Gabriella P. Sugerman^1^, Kelsey Varodom^1^, Manuel K. Rausch^1,3,4,5^, Janet Zoldan^1^, Hsin-Chin Yeh^1,6^, Sapun H. Parekh^1^*

*^1^Department of Biomedical Engineering, The University of Texas at Austin, 107 W Dean Keeton Street Stop C0800, Austin TX 78712, USA*

*^2^Centre for Biomedical Engineering, Indian Institute of Technology Delhi, New Delhi 110016, India*

*^3^Department of Aerospace Engineering and Engineering Mechanics, University of Texas at Austin, TX 78712*

*^4^Department of Mechanical Engineering, The University of Texas at Austin, TX 78712*

*^5^Oden Institute for Computational Engineering and Sciences, The University of Texas at Austin, TX 78712*

*^6^Texas Materials Institute, The University of Texas at Austin, Austin, TX, USA*

**
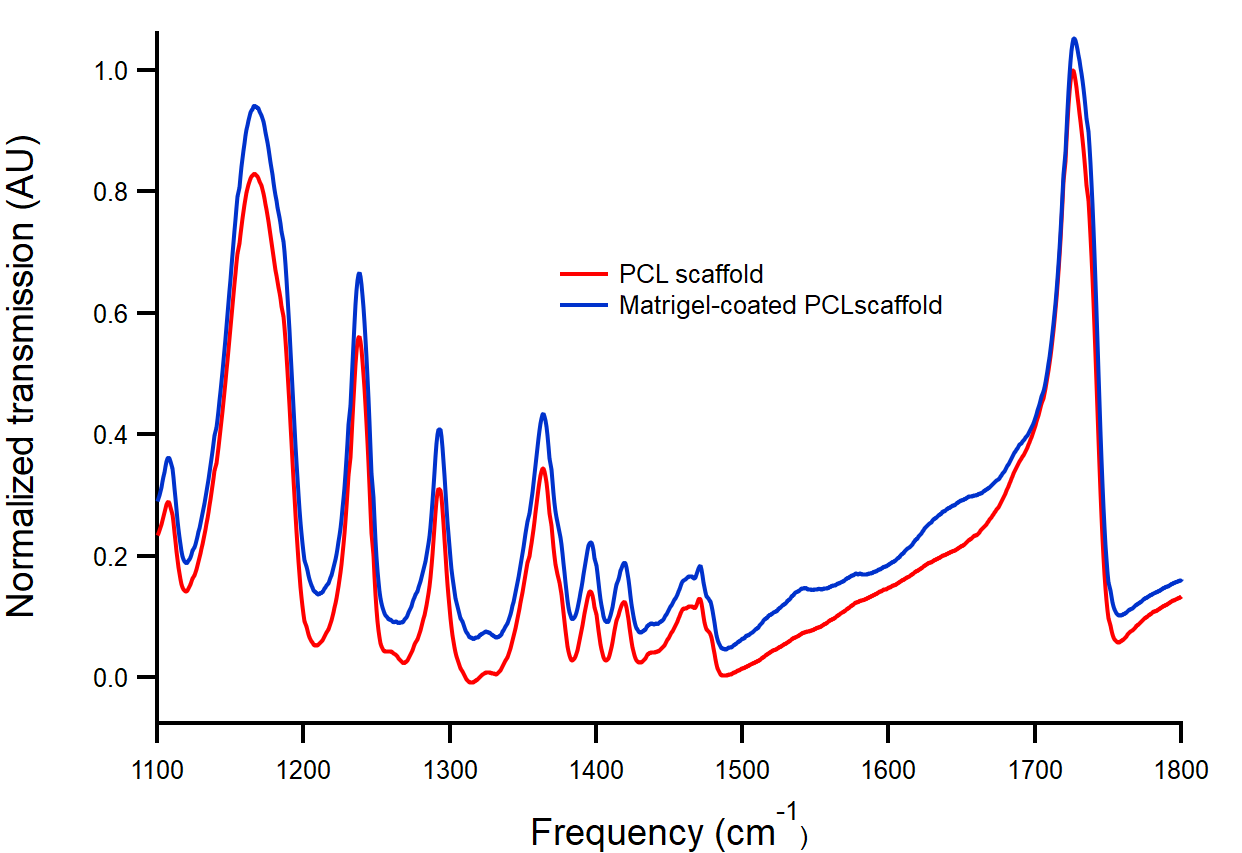
Correspondence:** sparekh@utexas.edu

**Figure S1**: Fourier transform infrared (FTIR) spectra of pure PCL (red) and Matrigel-coated PCL (blue) at a concentration 10-fold higher than used in the coating protocol. The shoulder of C=O stretching in the amide region indicated the presence of protein in the Matrigel-coated PCL scaffold (blue) compared to pure PCL fibers.


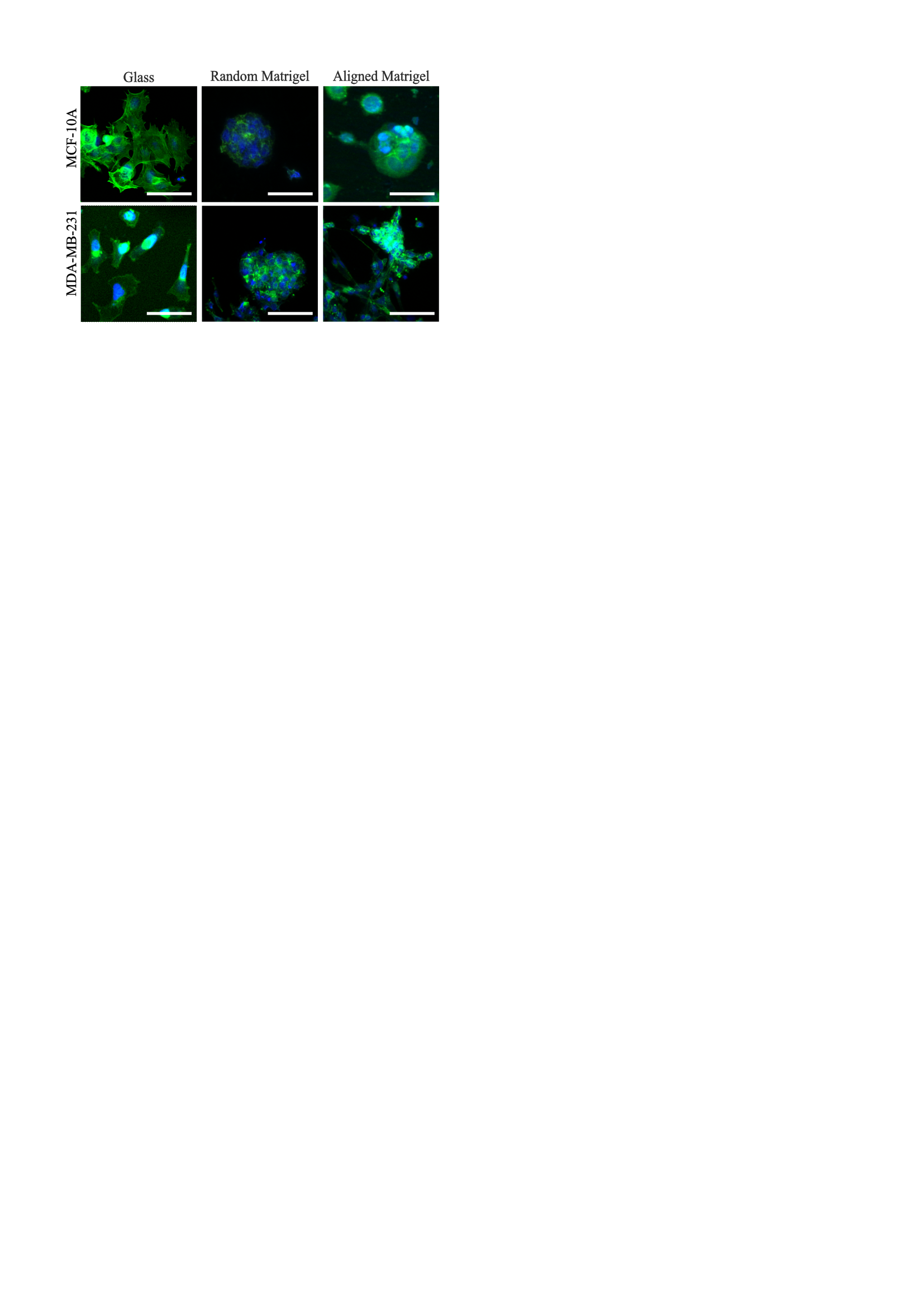
**Figure S2:** Fluorescence images of MCF-10A and MDA-MB-231 cells on respective substrates to demonstrate changes in cellular morphology. Green: Phalloidin (5 μM), Blue: DAPI nuclear stain (0.1 μg/mL). Scale: 50μm.

| **Primer Name** | **Sequence** |
| --- | --- |
| ACTB Forward | 5’-TGACGTGGACATCCGCAAAG-3’ |
| ACTB Reverse | 5’-CTGGAAGGTGGACAGCGAGG-3’ |
| Vimentin Forward | 5’-AGGCAAAGCAGGAGTCCACTGA-3’ |
| Vimetin Reverse | 5’-ATCTGGCGTTCCAGGGACTCAT-3’ |
| E-Cadherin Forward | 5’-ATTCTGATTCTGCTGCTCTTG-3’ |
| E-Cadherin Reverse | 5’-AGTCCTGGTCCTCTTCTCC-3’ |
| Snail Forward | 5’-ACTGCAACAAGGAATACCTCAG-3’ |
| Snail Reverse | 5’-GCACTGGTACTTCTTGACATCTG-3’ |
| CD44 Forward | 5’-AGAAGGTGTGGGCAGAAGAA-3’ |
| CD44 Reverse | 5'-AAATGCACCATTTCCTGAGA-3’ |
| MMP2 Forward | 5’-AGCTCCCGGAAAAGATTGATG-3’ |
| MMP2 Reverse | 5’-CAGGGTGCTGGCTGAGTAGAT-3’ |

**Table S1** Forward and reverse primers used in qPCR experiment.

**Supplementary Methods**

**Fourier transform infrared (FTIR) spectroscopy**

We used 1 mg/ml of Matrigel for coating PCL scaffolds for this measurement to ensure sufficient Matrigel was present to produce a detectable signal. A Vertex 70 spectrometer equipped with an MCT detector was used to collect the spectra at a frequency spacing of 2 cm^-1^. The raw FTIR spectra were collected and processed by a custom MATLAB script. Spectra were normalized at the maximum intensity in the C=O stretching (1740 cm^-1^).

**Actin stain nuclear staining**

**Materials Used for Immunofluorescence / immunocytochemistry**

Alexa Fluor 488 phalloidan (cat:A12379) was used to stain actin filaments and visualize cellular morphology. DAPI (4’,6-Diamidino-2-Phenylindole, Dihydrochloride) (cat: D1306) was used to counterstain the nucleus on respective biomaterial substrates.
